# Correlation Between Anti-Myelin Proteolipid Protein (PLP) Antibodies and Disease Severity in Multiple Sclerosis Patients With PLP Response-Permissive HLA Types

**DOI:** 10.3389/fimmu.2020.01891

**Published:** 2020-08-21

**Authors:** Judith M. Greer, Elisabeth Trifilieff, Michael P. Pender

**Affiliations:** ^1^UQ Centre for Clinical Research, The University of Queensland, Brisbane, QLD, Australia; ^2^Biopathologie de la Myéline, Neuroprotection et Stratégies Thérapeutiques, INSERM U1119, Université de Strasbourg, Faculté de Médecine, Strasbourg, France; ^3^Fédération de Médecine Translationnelle de Strasbourg (FMTS), Strasbourg, France; ^4^Faculty of Medicine, The University of Queensland, Brisbane, QLD, Australia; ^5^Department of Neurology, Royal Brisbane and Women's Hospital, Brisbane, QLD, Australia

**Keywords:** antibody, myelin proteolipid protein, multiple sclerosis, disease severity, HLA type

## Abstract

The most prominent pathological features of multiple sclerosis (MS) are demyelination and neurodegeneration. The exact pathogenesis of MS is unknown, but it is generally regarded as a T cell-mediated autoimmune disease. Increasing evidence, however, suggests that other components of the immune system, particularly B cells and antibodies, contribute to the cumulative CNS damage and worsening disability that characterize the disease course in many patients. We have previously described strongly elevated T cell reactivity to an extracellular domain of the most abundant CNS myelin protein, myelin proteolipid protein (PLP) in people with MS. The current paper addresses the question of whether this region of PLP is also a target of autoantibodies in MS. Here we show that serum levels of isotype-switched anti-PLP_181−230_ specific antibodies are significantly elevated in patients with MS compared to healthy individuals and patients with other neurological diseases. These anti-PLP_181−230_ antibodies can also live-label PLP-transfected cells, confirming that they can recognize native PLP expressed at the cell surface. Importantly, the antibodies are only elevated in patients who carry HLA molecules that allow strong T cell responses to PLP. In that subgroup of patients, there is a positive correlation between the levels of anti-PLP_181−230_ antibodies and the severity of MS. These results demonstrate that anti-PLP antibodies have potentially important roles to play in the pathogenesis of MS.

## Introduction

Multiple sclerosis (MS) is a chronic inflammatory demyelinating disease of the central nervous system (CNS), which affects about 2.5 million people worldwide. Although the exact pathogenesis of MS is unknown, it is predominantly regarded as a T cell-mediated autoimmune disease directed against myelin antigens. However, increasing evidence suggests that other components of the immune system, in particular B cells and antibodies, may contribute to the cumulative CNS damage and disability that characterize the disease course.

The single most consistent laboratory finding in MS is the presence of oligoclonal immunoglobulin G (IgG) bands in the cerebrospinal fluid (CSF) but not in the serum; these signify intrathecal antibody production, persist for the lifetime of the patient (1), and strongly suggest involvement of antibodies in the immunopathology of MS. Because CSF drains into the blood, and activated B cells can freely enter and exit the CNS, such antibodies could also enter the circulation. Numerous published studies have reported antibodies directed against myelin proteins (2–12) or other CNS antigens (13–18) in CSF or serum of MS patients, but only a small number of these studies have attempted to determine whether these antibodies are potentially pathogenic (3, 7, 15, 18). In some cases, antibodies in MS patients may be merely an epiphenomenon; however, there are multiple examples in the non-MS literature of how antibodies targeting CNS tissues can be directly pathogenic (reviewed in (19)), and the chance that antibodies present in at least some patients with MS are pathogenic is relatively high.

One question that is often raised is that of the relevance of serum antibodies to pathology in the CNS, since antibodies and complement are normally excluded from the CNS by the blood-brain barrier (BBB). However, activated B cells can easily cross the intact BBB (20) and differentiate into plasma cells which secrete antibody intrathecally, and during acute inflammatory episodes of MS there is increased permeability of the BBB, allowing entry of antibodies and complement components from the blood (21, 22). Thus, antibodies in both the serum and the CSF are of potential relevance in MS.

We have had a long-term interest in the role of immune T cell reactivity directed against the most abundant component of CNS myelin, myelin proteolipid protein (PLP), and have previously shown that T cell reactivity to PLP in MS is restricted by certain HLA types (23). Patients carrying these HLA types make stronger T cell responses to PLP peptides, especially those within the second extracellular loop of PLP (residues 181-230), and are more likely to develop lesions in the brainstem and cerebellum (23). Interestingly, we also found in a mouse model of MS that the sites in which lesions developed depended not only on T cells, but also on the presence or absence of anti-PLP antibodies against the same region of PLP (24, 25). PLP is present right throughout the lamellae of compact myelin, including the outermost loop (26), and monoclonal antibodies directed against epitopes within the two extracellular domains of PLP (encompassing residues 30–60 and 181–230) can live-label cultured oligodendrocytes (27), confirming that these PLP epitopes are exposed on the surface of oligodendrocytes, which makes them potential targets of demyelinating antibodies. Furthermore, a recent study has shown that monoclonal anti-PLP antibodies directed against these same extracellular epitopes of PLP can also label neurons (28), most likely through cross-reactivity between PLP and the related M6 family of molecules that are expressed on both neurons and oligodendrocytes. Since the most prominent pathological features of MS are demyelination and neurodegeneration, anti-PLP antibodies therefore have the potential to play a role in both of these aspects of MS.

Since strong antibody responses are reliant on T cell help, the work presented in this paper set out to determine if patients who carry HLA types that allow a strong T cell response to PLP also allow elevated antibody responses to PLP, and to determine if there is any relationship between levels of anti-PLP antibodies and disease severity in MS.

## Materials and Methods

### Patients and Controls

This study was approved by the Royal Brisbane and Women's Hospital Human Research Ethics Committee, and The University of Queensland Medical Research Ethics Committee. Up to 10 ml of blood was obtained from patients with MS [*n* = 146; 81 with relapsing-remitting MS (RR-MS), 38 with secondary progressive MS (SP-MS) and 27 with primary progressive MS (PP-MS)], a first demyelinating event suggestive of MS (clinically isolated syndrome (CIS); *n* = 40), patients with other CNS neurological disorders (OND; *n* = 42) and healthy individuals (*n* = 54). All patients with MS, apart from 4 PP-MS patients, met the 2010 revised McDonald criteria (29). None of the MS patients had received any immunosuppressant, immunomodulatory, or corticosteroid therapy in the 3 months prior to blood collection. Informed consent was obtained prior to blood collection. Five ml of the blood was allowed to clot at room temperature for 1–2 h, after which serum was collected. The other 5 ml of blood was used to extract genomic DNA for HLA typing. Demographics for individuals from whom blood was collected are shown in Table 1.

**Table 1:** Demographics of patients and controls.

**Group**	***n***	**% Female**	**Age median (range)**	**MS duration in years median (range)**	**MS severity score median (range)**
Healthy control	54	84.3%	38 (22–57)	n/a	n/a
MS (All)	146	80.1%	45 (19–72)	9 (0.25–31)	5.39 (0–9.88)
RR-MS	81	83.7%	37 (19–57)	5 (0.25–23)	4.44 (0–9.81)
SP-MS	38	89.5%	50.5 (27–72)	20 (3–31)	5.74 (1.29–9.63)
PP-MS	27	59.3%	52 (37–64)	11 (1–28)	8.04 (2.65–9.88)
OND	42	42.9%	46.5 (18–65)	n/a	n/a
CIS	40	75.0%	38 (20–56)	n/a	n/a

*n/a, not applicable*.

### HLA Typing

Genomic DNA was prepared using NucleoSpin Blood DNA extraction kits (Macherey-Nagel, Düren, Germany) as previously described (23). Dynal low and high resolution SSP kits (Dynal Biotech, Thermo Fisher, Australia) were used to type for HLA-DR, -DQA, and -DQB alleles, following the manufacturer's recommended protocols. Results for MS patients were reported to the 4 digit level, when it was able to be determined; however, alleles are grouped at the 2 digit level for the analyses.

### Human PLP and 50-mer PLP Peptide

Human brain tissue was obtained from the Queensland Brain Bank (part of the National Brain Bank Consortium) at the University of Queensland, and a total lipid extract was prepared using the method of Folch et al. (30). To prepare PLP, the total lipid extract was concentrated on a flash evaporator, precipitated with acetone, and the precipitate dried under nitrogen until all trace of the acetone was removed. The dried precipitate was then dissolved in a small volume of chloroform:methanol:acetic acid (2:0:0.03 v/v) and left at 4°C until the lipids (mainly cerebroside) rose to the top of the tube. The clear lower fraction was then subjected to gel chromatography through a 100 × 2.5 cm lipophilic LH-60 column at 1 atmosphere pressure, using chloroform:methanol (2:1) as the buffer. Elution of PLP from the column was monitored by measuring the absorbance at 280 nm. Fractions containing PLP were pooled and stored at 4°C in the dark until required. To convert PLP to a water soluble form, a small volume of PLP in chloroform:methanol in a shallow watch glass was diluted with an equal volume of chloroform:methanol:acetic acid (2:0:0.03 v/v), and then distilled deionized water (dH_2_O) was added, one drop at a time, under a constant stream of nitrogen gas. Once the liquid no longer turned cloudy when each drop of dH_2_O was added, 0.5 mL of dH_2_O was added, and the PLP was dialyzed against 3 changes of dH_2_O in 10,000 MW cutoff dialysis tubing. The protein concentration was determined using a bicinchoninic acid (BCA) assay (Pierce) and the sample was diluted to 1 mg/mL with dH_2_O. This water soluble form was kept at 4°C for no longer than 1 week.

The 50 mer PLP_181−230_ peptide containing 2 disulphide bonds (between cysteines residues at 200 and 219, and at 183 and 227), as occurs in the second extracellular loop of the native protein, was synthesized and checked for the presence of the correct two disulphide bonds as previously described (31). As this peptide has been shown to oxidize relatively rapidly, it was stored under nitrogen gas, and was prepared just prior to use by dissolving peptide in 0.2 M acetic acid to a concentration of 5 mg/mL.

### ELISA for Anti-PLP Antibodies

Since PLP precipitates out of solution in the presence of salts, it was diluted to 10 μg/mL in dH_2_O containing 25 μg/mL bovine serum albumin (BSA) and coated onto high protein binding Nunclon ELISA plates. Control wells were coated with 25 μg/mL BSA in dH_2_O. For the 50 mer PLP_181−230_ peptide, the 5 mg/mL stock solution was diluted to 5 μg/mL in bicarbonate buffer (pH 9.6) containing 25 μg/mL BSA for coating onto the ELISA plates. As negative control for the peptide-containing wells, ELISA plate wells were coated with the bicarbonate buffer containing 25 μg/mL BSA alone. Plates were blocked with 1% skimmed milk powder in PBS containing 0.05% Tween 20 (PBS-T-SM). Four dilutions (1/25, 1/50, 1/100 and 1/200) of a control serum sample (which was a pool of 5 MS sera with moderate to high reactivity in preliminary assays) diluted in PBS-T-SM were used on each plate to serve as a positive control to ensure consistency of peptide coating and to normalize results from one plate to another (see below). Each test serum sample was tested at a dilution of 1:40 in PBS-T-SM on three wells coated with the BSA alone, and three wells coated with the BSA + PLP_181−230_ peptide. After 2 h at room temperature, plates were washed with PBS-T, and an alkaline phosphatase-conjugated anti-human polyvalent Ig secondary antibody was added to all wells of the plate for 2 h at room temperature. After washing 5 times with PBS-T and once with dH_2_O, the substrate p-nitrophenyl phosphate (pNPP; Sigma) was added to each well, incubated for 15 min, and the reaction was then stopped with 3 N NaOH. The absorbance at 405 nm was read on a Tecan Spark 10 M Multimode plate reader. The absorbance of the wells containing BSA alone was subtracted from the absorbance of the wells containing BSA + PLP_181−230_ for each sample, to give the PLP-specific absorbance. A semi-log XY standard curve was drawn using the 4 dilutions of the positive control serum sample on the x axis (log scale) and their absorbance values on the y axis, and the equation of the curve determined (Y = slope x log_10_(X) + Y intercept). From that equation, *X* values for each test serum sample were calculated. The absorbance of the 1/100 dilution was normalized to 1 absorbance unit, which changes the *Y* intercept, but not the slope of the curve. From the equation of this normalized curve, the normalized *Y* values of the test samples were obtained (i.e., normalized PLP_181−230_-specific absorbance values).

### Isotyping

For isotyping of samples, the ELISA was repeated on serum samples that showed a detectable level of PLP_181−230_ specific antibodies above the level of the 75% percentile of the healthy individuals. In this case, however, the secondary antibodies used were horse radish peroxidase (HRP) conjugated mouse antibodies specific for human IgG1, IgG2, IgG3, IgG4, or IgM antibodies (clones HP6070, HP6014, HP6047, HP6023 and HP6083, respectively, all from Invitrogen), which were used instead of the alkaline phosphatase-conjugated polyvalent human Ig antibody above. To detect the HRP-conjugated secondary antibodies, plates were incubated with o-phenylenediamine dihydrochloride (OPD) substrate (Sigma) for 15 min at room temperature, stopped with 2.5 M sulphuric acid, and the absorbance was read at 490 nm. Each sample was tested in triplicate on wells coated just with BSA and on wells coated with BSA + PLP_181−230_ for the presence of each different IgG or IgM isotype. Data were reported as the isotype of antibody that gave the strongest PLP_181−230_ specific response.

### Immunolabelling of PLP-Expressing CHO Cells

A plasmid encoding wild-type human *PLP1* coupled to an mCherry tag was constructed as previously described (32). Dissociated CHO-K1 cells (10^6^ cells in PBS) were transiently transfected via electroporation with 2 ng of the plasmid using the Amaxa II transfection device (Lonza, Basel, Switzerland), following the manufacturer's recommended protocol. The transfected cells were plated in 8 well-chamber slides at a concentration of 10^5^ cells/well. Two days following transfection, the cells were live-labeled with 1/20 dilution of patient serum at room temperature for 1 h. After 3 washes with PBS containing 2% fetal calf serum and 0.05% azide (PBS azide wash), cells were labeled with FITC-conjugated rabbit anti-human Igs (Dako, Agilent, Santa Clara, USA) at room temperature for 1 h. Slides were washed 3 times in PBS azide wash, and the chambers were removed. Finally, the slides were incubated for 15 min with PBS azide wash containing 1/30,000 dilution of DAPI, and then coverslips were mounted on the slides. Slides were viewed using a Zeiss Axio Imager M1 microscope fitted with an Axiocam 503 camera, and images acquired using Zen software (Zeiss).

### Statistical Analysis

Statistical analyses were done using GraphPad Prism v 8.2.1. Data were first checked to determine if they were normally distributed. If so, 3 or more groups were compared using ANOVA, with Bonferroni correction for multiple comparisons. Data in this case are presented as mean ± SE of the mean. If data were not normally distributed, then the Kruskal-Wallis test with Dunn's multiple comparison test was used to determine statistical significance. In that case, data are presented as median and interquartile range. For correlations, since the data were from a non-parametric distribution, the Spearman correlation coefficient (ρ) was determined.

## Results

### Comparing the Reproducibility of ELISA Results Using Human PLP or the PLP_181−230_ Peptide

Whole PLP has been used as an antigen in various T cell and antibody studies, with varying levels of reproducibility and success, as previously discussed (33). One reason for this variability is the extreme hydrophobicity of PLP. It is difficult to get PLP into a water-soluble form, and, even then, the presence of salts tends to make it precipitate out of solution. We have previously shown that the major T cell response against PLP is directed against two overlapping peptides, PLP_184−199_ and PLP_190−209_, within the second extracellular loop of PLP (PLP_181−210_), and that there is also an elevated antibody response to these peptides (23). However, the second extracellular loop of PLP normally contains 2 disulphide bonds, which would potentially produce conformational epitopes for antibodies that would not be able to be detected using the 2 overlapping peptides above. Therefore, we decided first to compare the reproducibility of antibody assays utilizing whole human PLP or a 50-mer PLP peptide covering this second extracellular loop, and synthesized with 2 disulphide bonds, to mimic the structure of PLP in the myelin membrane. Initially, serum samples from 82 MS patients were tested for reactivity against whole human PLP or the 50-mer peptide, with each sample tested in 3 independent assays. Each sample was scored as a positive (absorbance > 0.2 units) or negative (absorbance ≤ 0.2 units) response to human PLP and to PLP_181−230_, and the proportion of samples that scored all positive or all negative in the 3 independent assays was determined. For whole human PLP, the same result was obtained in all 3 replicate assays only 48.8% of the time. In contrast, using the PLP_181−230_ peptide, the same result was obtained in all 3 replicates 87.8% of the time, which was a marked improvement over the result with whole human PLP. In cases where a sample showed a positive response to the whole PLP protein, it also gave a positive result to the PLP_181−230_ peptide in 92.9% of cases. In contrast, if the response to the whole protein was negative, the response to the peptide was negative in only 70% of cases. Thus, we believe that the peptide has better sensitivity for detection of the anti-PLP antibodies.

### Antibodies That Bind to the PLP_181−230_ Peptide in ELISA Can Also Live-Label PLP-Transfected Cells

**Figure 1:**
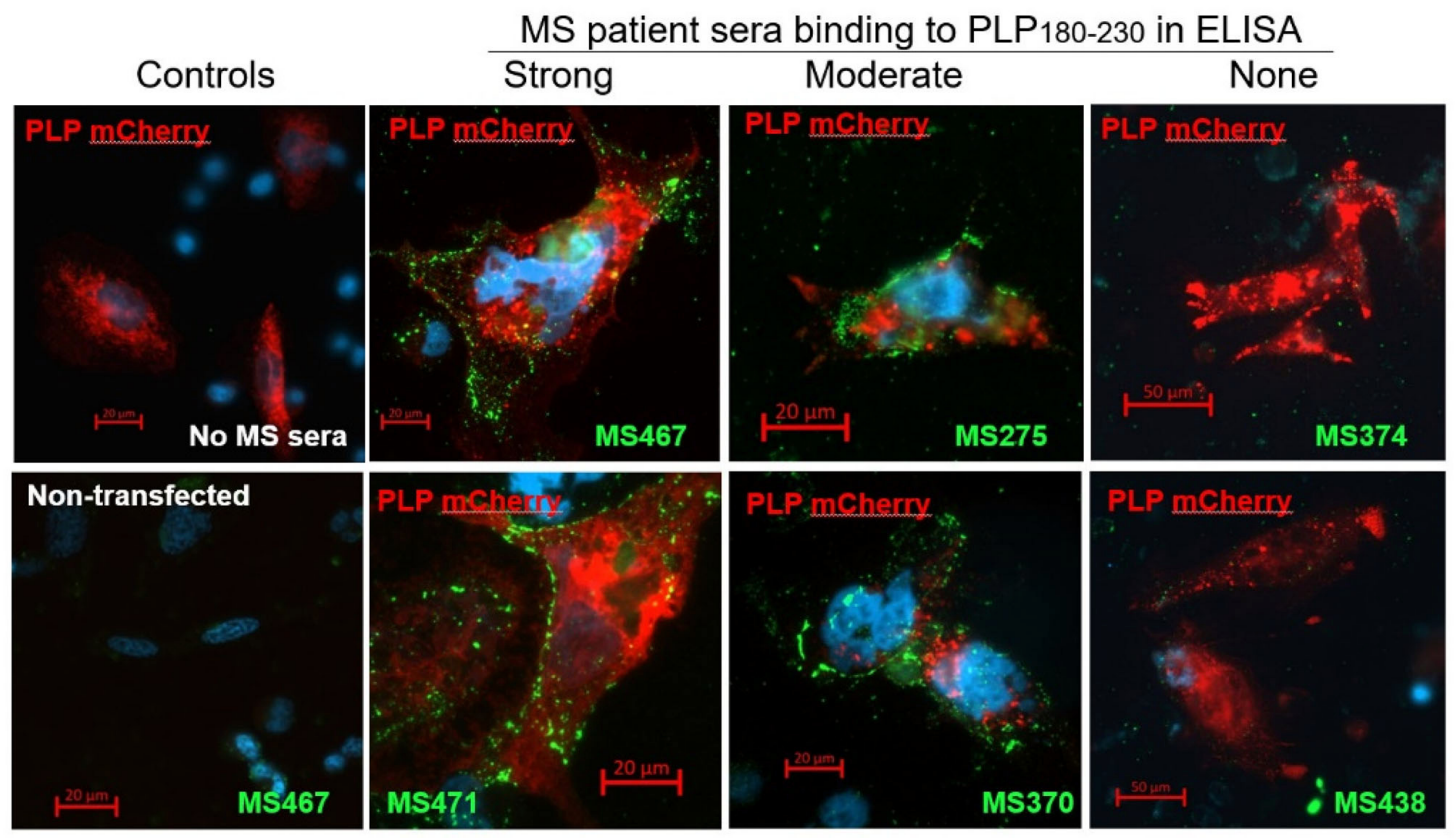
CHO cells transiently transfected with an mCherry-PLP construct (so that cells expressing PLP fluoresce red) were live-labeled using MS patient sera that showed high (MS467 and MS471), moderate (MS275 and MS370) or no (MS374 and MS438) binding to the PLP_181−230_ peptide in ELISA assays. Binding of patient sera to the transfected CHO cells was revealed by addition of a FITC-labeled anti-human Ig secondary antibody (green). Controls included wells with no MS sera added (top left image) and non-transfected cells in the wells to which MS sera were added (bottom left image showing non-transfected cells in the well in which MS467 serum was added). MS sera that showed high or moderate binding to the PLP_181−230_ peptide in ELISA assays labeled the membranes of PLP-transfected CHO, whereas sera that showed no anti-PLP_181−230_ binding by ELISA did not label the PLP-transfected CHO cells.

To confirm that the anti-PLP_181−230_ antibodies detected in the ELISA are also able to recognize whole PLP expressed in the cell membrane, CHO cells were transiently transfected with a mCherry-tagged PLP-expressing plasmid, and live-labeled 2 days later with sera from patients who showed strong, moderate, or no reactivity in the ELISA. There was a good correlation between the anti-PLP_181−230_ reactivity of the serum samples in the ELISA and their ability to live-label PLP-transfected cells (Figure 1). The antibodies from patients with high or moderate antibody levels measured by ELISA showed strong membrane staining of the PLP-transfected cells, but not of non-transfected cells in the same slide, whereas sera that showed no anti-PLP_181−230_ binding by ELISA did not label the PLP-transfected CHO cells. Therefore, the remainder of the assays in this paper were done using the PLP_181−230_ peptide in ELISA for determining levels of anti-PLP antibodies.

### Levels of Isotype-Switched Autoantibodies Specific for PLP_181−230_ Are Increased in Patients With MS

**Figure 2:**
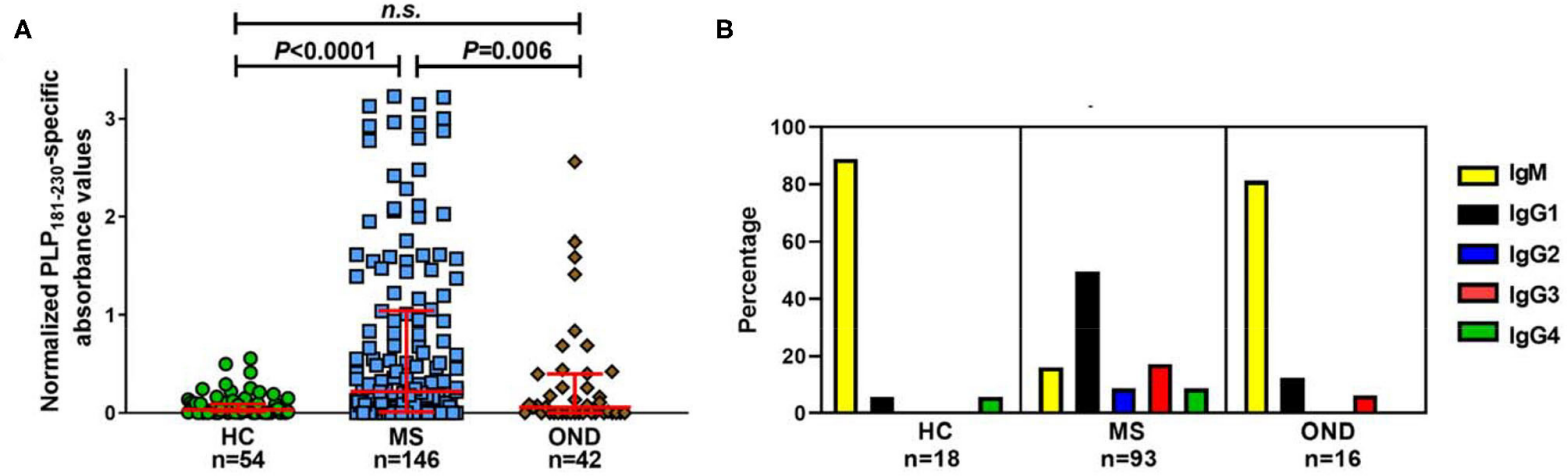
Significantly elevated levels of isotype-switched anti-PLP antibodies are present in MS patients. **(A)** Reactivity in ELISA of healthy individuals' (HC), MS and OND patients' sera (all tested at 1/40 dilution) to PLP_181−230_, synthesized with 2 disulphide bonds to mimic the structure of the second extracellular loop of PLP. Results are presented as the normalized PLP_181−230_-specific absorbance values. Analyzed using Kruskal–Wallis test with Dunn's multiple comparison test. **(B)** Percentage of samples of each isotype from individuals with elevated (at or above the 75th percentile of the HC group) antibodies reactive to PLP. n.s., not significant.

Initial studies investigated the levels of antibodies specific for PLP_181−230_ in patients with MS, OND patients and healthy individuals. There was a significant increase in the levels of anti-PLP_181−230_ antibodies in MS patients, compared to the OND patients and healthy individuals (Figure 2A). Some OND patients did show elevated levels of anti-PLP_181−230_ antibodies. The OND patients with an elevated antibody response to PLP in the top quartile of the OND group had a variety of CNS disorders, including epilepsy (3 patients), stroke (3 patients), and one each of CNS tumor and idiopathic intracranial hypertension.

Next, we looked at the isotype of the anti-PLP_181−230_ antibodies. Sera from individuals who showed levels of antibodies at or higher than the 75th percentile of the healthy individual group were tested to determine the primary isotype of antibodies reacting with the PLP peptide. This included samples from 18 healthy individuals, 93 patients with MS, and 16 OND patients. Of interest, almost all of the positive samples from OND patients (13/16) and healthy individuals (16/18) contained IgM anti-PLP_181−230_ antibodies only (Figure 2B). In contrast, the majority of the positive samples from MS patients contained isotype-switched anti-PLP_181−230_ antibodies. IgG1 was the most common isotype for anti-PLP_181−230_ antibodies from MS patients; however, several patients had high levels of IgG2, IgG3, or IgG4 antibodies, suggesting that the microenvironment in which the anti-PLP antibody producing B cells mature and class-switch can vary from patient to patient. The percentage of MS patients who had isotype-switched anti-PLP antibodies to any IgG subtype (83.9%) was highly significantly different from that of healthy controls (11.1%; *P* = 1.8 × 10^−9^) and that of OND patients (18.8%; *P* = 2.0 × 10^−7^) by χ^2^ with Yates' correction.

### Anti-PLP Antibodies Are Produced Throughout the Course of MS

**Figure 3:**
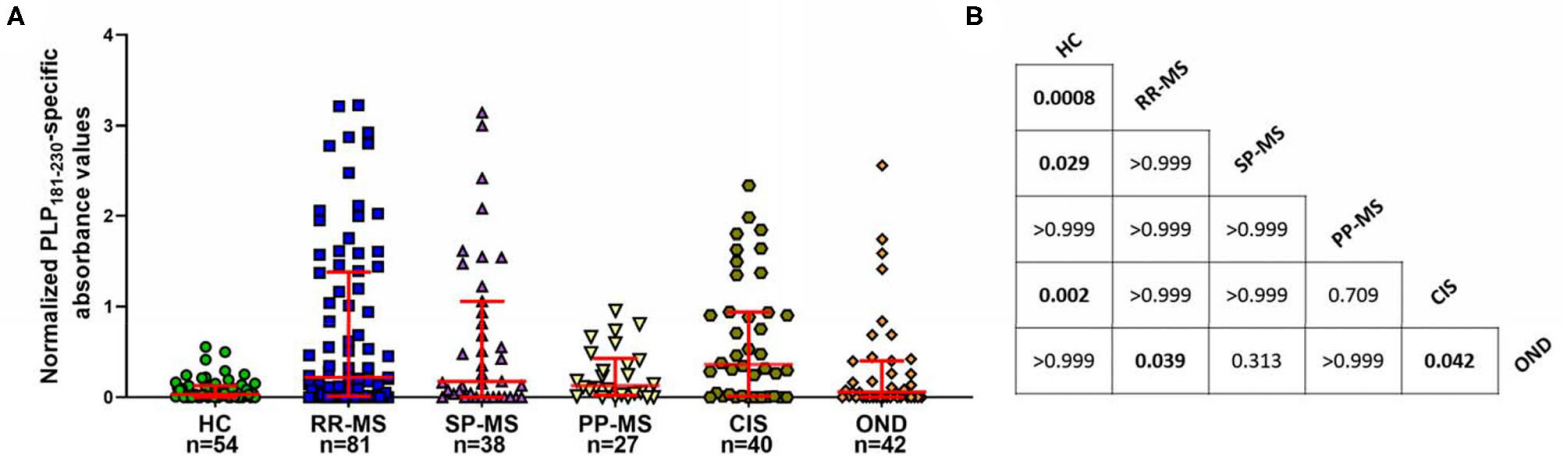
**(A)** Comparison of anti-PLP_181−230_ antibody levels in the different MS subgroups and in patients with CIS. PP-MS patients have lower levels of anti-PLP_181−230_ antibodies than do other groups of MS patients or individuals with CIS. **(B)** Chart showing the *P*-values for comparison of all groups shown in **(A)** Significant differences are highlighted in bold. Analyzed using Kruskal-Wallis test with Dunn's multiple comparison test.

Next, we investigated if there were differences between patients with different disease courses, and also if CIS patients had elevated levels of anti-PLP_181−230_ antibodies. As shown in Figure 3, the levels of antibodies were elevated more in patients with RR-MS and SP-MS than in PP-MS, although this was not statistically significance. Most interestingly, approximately half of the CIS patients showed elevated antibody responses to PLP_181−230_, suggesting that these antibodies are present from the early stages of disease. The anti-PLP_181−230_ antibody levels were significantly different in RR-MS patients and CIS patients compared to both healthy individuals and OND patients, whereas the SP-MS patient antibody levels were only significantly different to healthy individuals, and PP-MS patient antibody levels were not significantly different to either the healthy individuals or OND patients.

### Patients Carrying Certain HLA Types Are Much More Likely to Produce Elevated Levels of Anti-PLP Antibodies

Previously we have described that certain HLA class II alleles in particular alleles within the HLA-DRB1^*^04, DRB1^*^07 or DRB1^*^13 families, and the DQ8 serotype (which is in strong linkage disequilibrium with HLA-DRB1^*^04 alleles) correlate strongly with elevated T cell reactivity to peptides from the 184-209 region of PLP (23). The same PLP peptides appear to bind minimally to the MS-related HLA molecule DRB1^*^15:01 and to DRB1^*^03, which is also found commonly in Caucasian MS patients. Furthermore, they cannot induce disease in HLA transgenic mice expressing these alleles. However, PLP_175−194_ can induce demyelinating disease in mice transgenic for HLA-DQB1^*^06:02, which forms the β chain of the DQ6 type that is in strong linkage disequilibrium with DRB1^*^15:01 (34). We reasoned that patients showing isotype-switched anti-PLP_181−230_ antibodies might be more likely to also be able to make a strong T cell response to peptides in this region of PLP, as T cell help is likely required for the isotype switching to occur.

**Figure 4:**
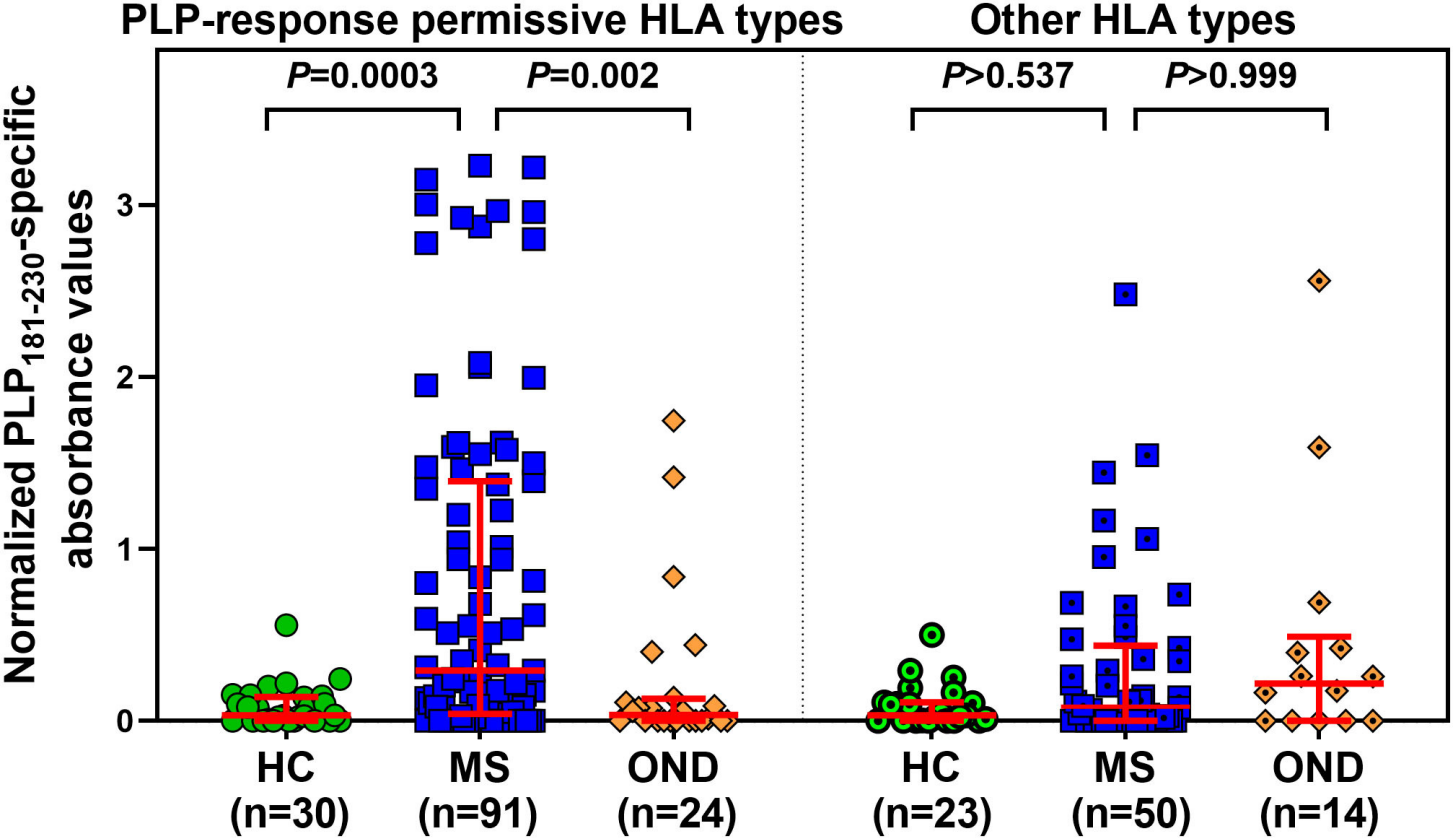
Comparison of anti-PLP_181−230_ antibody reactivity in individuals carrying PLP response-permissive HLA types vs. other HLA types. There were no significant differences between HC, MS, and OND groups in the patients carrying other HLA types.

Levels of anti-PLP_181−230_ antibodies were assessed on the basis of whether patient had HLA genotypes containing DRB1^*^03, DRB1^*^04, DRB1^*^07, DRB1^*^13, or DRB1^*^15:01, either in the presence or absence of DRB1^*^15:01 as the second allele (in order to assess whether the DRB1^*^15:01 was contributing to the response). Patients who carried none of these alleles are listed as “other, other” genotype. As shown in Table 2, the responses of samples from 146 MS patients and 20 CIS patients from whom HLA typing was available showed that patients carrying HLA- DRB1^*^04, DRB1^*^07, or DRB1^*^13 were significantly elevated compared to the “other, other” genotype, and that this was not dependent on the presence of DRB1^*^15:01, as there were no significant differences between (e.g.,) people with DRB1^*^04, DRB1^*^15:01 vs. DRB1^*^04, other genotype. Interestingly, the only patients who showed a difference between the presence and absence of DRB1^*^15:01 as the second allele were individuals who were homozygous for DRB1^*^15:01, who had elevated levels of anti-PLP_181−230_ antibodies compared to those who had DRB1^*^15:01 and an allele other than DRB1^*^03, DRB1^*^04, DRB1^*^07, DRB1^*^13, or DRB1^*^15:01. It is likely that the elevated response in DRB1^*^15:01 homozygous individuals is due to increased expression of HLA-DQ6 in these patients, as studies in patients with narcolepsy have shown that allelic dosage of DQB1^*^06:02 is transmitted into significant changes in HLA-DQ6 heterodimer availability (35). Thus, these results show that PLP response permissive HLA types include DRB1^*^04, DRB1^*^07, DRB1^*^13, and homozygosity for DRB1^*^15:01. When the ELISA data from MS patients and controls shown in Figure 2A was assessed on the basis of carriage of these PLP response-permissive HLA types, there was still a highly significant difference between MS patients and controls for the PLP response-permissive HLA types, but not for those individuals who do not carry these HLA types (Figure 4). Most of the MS patients who did not carry the typical PLP response-permissive HLA types, but who showed higher levels of reactivity to PLP, carried DRB1^*^11 alleles. DRB1^*^11 is usually in linkage disequilibrium with DQ7 (DQB1^*^03), which is also expressed by many patients who carry DR4 or DR13 alleles. It may be that in some MS patients, the HLA restriction is actually through the HLA-DQ rather than HLA-DR molecules. It is notable that 2 OND patients who had high levels of anti-PLP antibodies but who did not carry typical PLP response-permissive HLA types both carried DRB1^*^10 alleles (which occur very infrequently in MS patients – only one MS patient in this study carried DRB1^*^10). Therefore, certain DQ and DRB1^*^10 alleles may represent additional PLP response-permissive HLA molecules, but that remains to be proven.

**Table 2:** Relationship between HLA genotype of MS patients and antibody response to PLP_181−230_.

**HLA-DRB1 genotype**	***n***	**Response to PLP_**181−230**_ median (IQR)^¶^**	*P* vs. “other, other” genotype^*^
03, 15:01 03, other	13 13	0.027 (0–0.296) 0.076 (0–0.323) *P*_(03,15:01 vs. 03,other)_ = 0.930	0.608 0.550
04, 15:01 04, other	14 29	0.467 (0.050–1.945) 0.181 (0.064–1.467) *P*_(04,15:01 vs. 04,other)_ = 0.528	**0.007** **0.013**
07, 15:01 07, other	12 14	0.709 (0.109–1.271) 0.640 (0.060-2.834) *P*_(07,15:01 vs. 07,other)_ = 0.981	**0.011** **0.020**
13, 15:01 13, other	11 18	0.835 (0.104–1.951) 0.752 (0.087–1.480) *P*_(13,15:01 vs. 13,other)_ = 0.811	**0.006** **0.009**
15:01, 15:01 15:01, other	19 18	0.884 (0.121–1.224) 0.108 (0.025–0.379) *P*_(15:01,15:01 vs. 15:01,other)_ = **0.026**	**0.006** 0.281
Other, other	10	0.003 (0–0.243)	**–**

“Other” indicates alleles other than DRB1^*^03, DRB1^*^04, DRB1^*^07, DRB1^*^13 or DRB1^*^15:01.

¶ *The P-value shown below the median and IQR for each pair of genotypes shows the significance of the comparison between those two groups*.

* *Significant P-values are indicated by bold type*.

### Levels of Antibody Responses to PLP_181−230_ Correlate With Disease Severity in RR-MS and SP-MS Patients Carrying PLP Response-Permissive HLA Types

**Figure 5:**
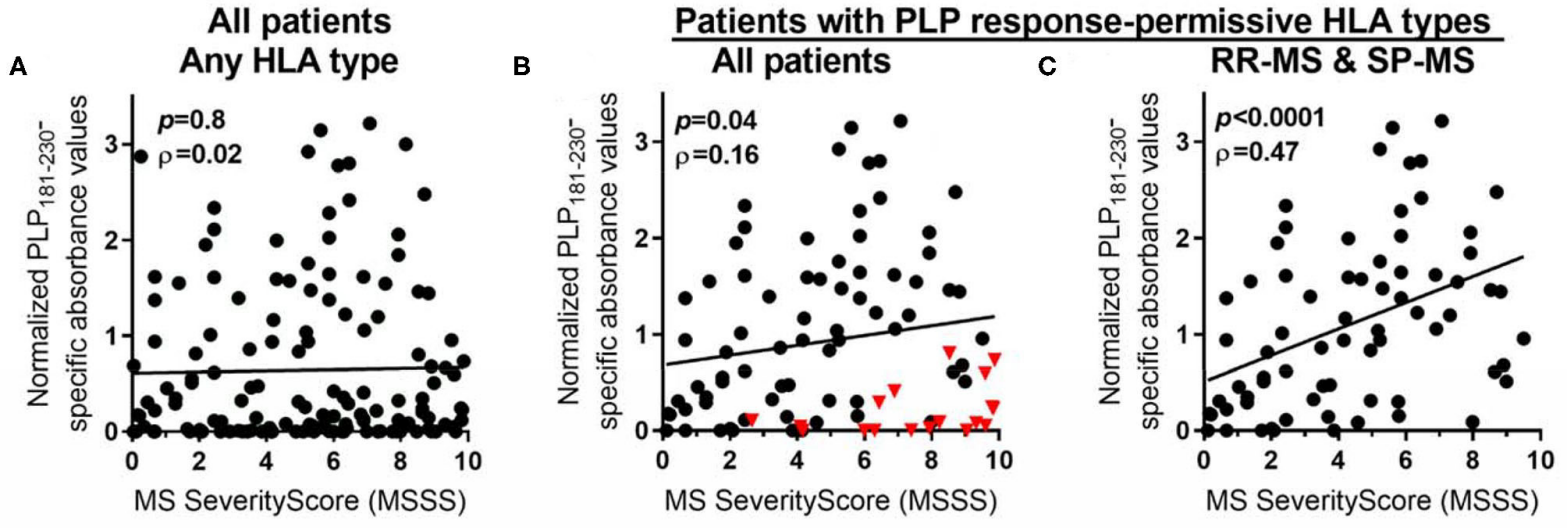
Spearman rank correlation between levels of anti-PLP antibodies (as determined in Figure 1) and MSSS in: **(A)** all MS patients, irrespective of their HLA type; **(B)** all MS patients who carry PLP response-permissive HLA types (red triangles indicate PP-MS patients); and **(C)** RR-MS and SP-MS patients who carry PLP response-permissive HLA types. The *P*-value for each correlation and the Spearman ρ (rho) value are shown in each graph.

The levels of anti-PLP antibodies were also correlated with the MS Severity Score (MSSS) (36), which uses disability and disease duration to rate disease severity. When all patients were included in the analysis, there was no significant correlation between the levels of anti-PLP antibodies and the MSSS (Figure 5A). However, as shown above, certain HLA types correlate with elevated levels of anti-PLP_181−230_ antibodies. We therefore also analyzed the correlation between anti-PLP antibody levels in patients carrying the PLP response-permissive HLA types and the MSSS: in this group there was a significant correlation between the level of anti-PLP antibody and the MSSS (*P* = 0.04; Figure 5B). Interestingly, the majority of patients with low levels of anti-PLP antibody but high MSSS in this group had PP-MS (indicated by the red triangles in Figure 5B). When only RR-MS and SP-MS patients were included in the analysis, the correlation was much stronger (*P* < 0.0001; Figure 5C). The ρ value is relatively low (0.46); however, this may reflect differences in the disease state at the time of testing, with patients who were tested while in remission typically showing lower levels of antibody than those tested during an attack of MS. This data demonstrates that, in RR-MS and SP-MS patients who carry HLA types that allow autoreactivity to PLP to occur, anti-PLP antibodies show a correlation with disease severity. Furthermore, the finding that anti-PLP antibodies do not correlate with disease severity in patients with PP-MS would support the idea that the disease process in PP-MS is different from that seen in RR-MS/SP-MS.

## Discussion

In this paper, we have demonstrated that isotype-switched antibodies targeting PLP_181−230_ are significantly elevated in the serum of patients with RR-MS and SP-MS, particularly in those carrying PLP response-permissive HLA types, and that there is a positive correlation between the levels of antibody and disease severity in these patients.

The use of the PLP_181−230_ peptide, synthesized so as to fold with 2 disulphide bonds as occurs in PLP expressed at the oligodendrocyte cell membrane, significantly improved the reproducibility of the anti-PLP antibody results, compared to assays done using whole human PLP. A rabbit serum, raised against this 50-mer peptide, has been shown to be able to live label rat oligodendrocytes (37), in a pattern reminiscent of that seen when cells were labeled with the O10 monoclonal antibody, which recognizes a conformation-dependent epitope of the second extracellular loop of PLP (38). The PLP amino acid sequence is identical between human, mouse and rats, and therefore the fact that antiserum raised against the PLP_181−230_ epitope could recognize rat oligodendrocytes suggested that antibodies of this specificity could also recognize human PLP. We have now shown in this paper than CHO cells, transfected to express human PLP, can be live labeled with antibodies that show elevated levels of reactivity to PLP_181−230_ in the ELISA assays, suggesting that the antibodies of this specificity could be of potential functional relevance in humans.

Importantly for the synthesis of the PLP_181−230_ peptide for testing of human antibody responses, this region of PLP contains two residues that differ between the human and bovine sequences, namely at residues 188 and 198. Many early studies on immune reactivity to PLP used bovine PLP as the antigen, and results from those studies were not particularly reproducible (33). However, it is not unexpected that difficulties arise when using whole PLP as an antigen in an ELISA or in other immunological assays, as, owing to its hydrophobicity and intolerance for solutions containing salt, the whole PLP molecule is not a suitable molecule for most of these assays. We have previously shown that there are elevated levels of antibodies against PLP_184−199_ and PLP_190−209_ in patients with MS compared to healthy individuals and OND patients (23), although the levels of antibodies were generally lower than those seen using the PLP_181−230_ peptide. We suggest that this is due to the improved ability to detect antibodies against conformationally relevant epitopes of the second extracellular loop when using the PLP_181−230_ peptide.

The finding that most MS patients had isotype-switched anti-PLP_181−230_ antibodies, whereas those healthy individuals or OND patients with detectable levels of anti-PLP_181−230_ antibodies generally had antibodies of the IgM isotype, suggests the presence of a PLP-driven immune process occurring in the MS patients. Testing of CSF for the presence of PLP-specific antibodies was not done in the current study, but will be a focus of future investigations.

In previous work, we have identified that ~40–50% of MS patients can show elevated T cell proliferative responses to various epitopes of PLP, but that the response is directed against the second extracellular loop of PLP in most patients (23, 39, 40). Similarly in experimental animals, overlapping epitopes within the second extracellular loop of PLP form a cluster of immunogenic and encephalitogenic peptides for mice from many genetic backgrounds (41). We would therefore suggest that this second extracellular loop of PLP is the most likely target of disease-relevant autoreactivity in MS.

It is of interest to note that nearly half of the patients with a CIS suggestive of MS had elevated levels of anti-PLP_181−230_ antibodies. There may therefore be some predictive power in studying these antibodies in early MS. However, as shown in this paper, the HLA type of the patients (and therefore the potential to develop strong T cell responses to PLP) also plays a role in whether or not antibodies develop. Previously we have shown that in a patient who carries a PLP response-permissive HLA type, highly increased numbers of PLP-specific T cells could be detected in both the blood and the cerebrospinal fluid (CSF) right from the earliest stage of disease (23). Therefore, it is likely that any predictive modeling of disease severity from early timepoints, based on the presence of anti-PLP antibodies, would need to take into account the HLA type of the patients.

There are many ways in which anti-PLP antibodies, particularly those targeting epitopes on the extracellular surfaces of oligodendrocytes or myelin, could potentially have an impact in MS, including mechanisms such as complement-mediated lysis, antibody mediated cell cytotoxicity, modulation of cell architecture, opsonization of myelin or myelin debris leading to increased activation of phagocytic cells (19). In a C3H/HeJ mouse model in which demyelinating disease can be induced by immunization with PLP_190−209_, we have previously reported that mice that can make a T cell response, but not an antibody response, develop lesions in the brainstem, but not in the cerebellum, whereas in the presence of both T cells and antibodies specific for PLP_190−209_ there is development of lesions in both the brainstem and the cerebellum (23, 25). This suggests either that the anti-PLP antibodies can shift the sites of lesions, or alternatively that there is more severe disease when both the T cells and antibodies are present.

A recent study using several monoclonal antibodies specific for the first and second extracellular domains of PLP also suggests that antibodies against these regions of PLP might be able to cause damage to neurons (28). This study showed that the antibodies, specific for either PLP_50−69_ or PLP_178−191_, could bind to cell surface proteins on neurons in human brain, and that, *in vitro*, the antibodies could inhibit neuronal differentiation and outgrowth of neurites. Preliminary findings suggested that the cross-reactivity between the anti-PLP antibodies and the neurons could be via the M6 proteins. M6a and M6b are glycoproteins belonging to the same gene family as PLP. They are involved in neuronal and axonal guidance, in an integrin-dependent fashion (42, 43). M6a is only expressed on neurons, but some M6b isoforms are also expressed by oligodendrocytes (44). The PLP_181−230_ region has 50% sequence similarity to that of M6a and 72% similarity to M6b. Therefore, there is the potential that anti-PLP_181−230_ specific antibodies produced by MS patients could bind to neurons and cause damage to them. Such effects could contribute to the apparent correlation between disease severity and the levels of anti-PLP_181−230_ specific antibodies, as disease severity is, in large part, caused by underlying irreversible damage to neurons. Interestingly, PP-MS is usually thought to involve a greater degree of irreversible axonal damage and brain atrophy than are other forms of MS. However, we did not find any relationship between severity of PP-MS and levels of anti-PLP_181−230_ antibodies. We have previously reported that patients with PP-MS carry a different complement of HLA alleles to those found in patients with RR-MS or SP-MS, in particular they are more likely to carry alleles with a negatively charged glutamic acid residue in pocket 4 of the antigen-binding site of the HLA-DR molecules (45). In addition, PP-MS patients show significantly lower T cell proliferation in response to PLP_184−199_ or PLP_190−209_ peptides compared to RR-MS and SP-MS patients (39). It is likely that these differences in the HLA molecules carried by PP-MS patients affects their ability to make effective antibody responses to PLP_181−230_.

Overall, the results of this study suggest that anti-PLP_181−230_ antibodies have potentially important roles to play in the pathogenesis of MS, particularly in patients with RR-MS and SP-MS.

## Data Availability Statement

The datasets generated for this study are available on request to the corresponding author.

## Ethics Statement

The studies involving human participants were reviewed and approved by Royal Brisbane and Women's Hospital Human Research Ethics Committee and The University of Queensland Medical Research Ethics Committee. The patients/participants provided their written informed consent to participate in this study.

## Author Contributions

JG and MP contributed to conception and design of the study. JG extracted human PLP, performed all antibody experiments, and wrote the first draft of the manuscript. ET designed and synthesized the PLP peptide. MP recruited patients for the study and did clinical assessments. All authors contributed to manuscript revision, and read and approved the submitted version.

## Conflict of Interest

The authors declare that the research was conducted in the absence of any commercial or financial relationships that could be construed as a potential conflict of interest.
